# Myometrial mechanotransduction and the timing of parturition: ion-channel pathways linking uterine stretch, calcium signaling, and inflammation

**DOI:** 10.3389/fphys.2026.1901359

**Published:** 2026-07-29

**Authors:** Xinyu Han, Tianqiang Wu, Xinxin Hu

**Affiliations:** Wenzhou Hospital of Traditional Chinese Medicine, Wenzhou, China

**Keywords:** calcium signaling, mechanotransduction, myometrium, parturition, PIEZO1, preterm labor, TRPV4, uterine stretch

## Abstract

Mechanical loading is a defining feature of pregnancy, yet its integration with ion-channel physiology in the myometrium remains incompletely synthesized. This Review examines how uterine stretch, overdistension, oxytocin-associated signaling, extracellular-matrix context, and inflammatory cues are translated into Ca²^+^ entry, membrane excitability, inflammatory amplification, and contraction timing in the pregnant myometrium. It distinguishes candidate direct mechanosensors from mechanically regulated Ca²^+^/inflammatory coupling pathways and from adjacent excitability regulators, with the strongest human myometrial evidence currently centered on PIEZO1 and setting-limited TRPV4 signaling. It further separates physiologic term parturition from overdistension-associated premature activation and infection/inflammation-associated preterm birth, which can share downstream modules without being mechanistically interchangeable. Finally, it defines the experimental and translational boundaries that remain to be resolved before channel-centered mechanisms can be interpreted as pharmacologic or clinical intervention strategies.

## 1 Introduction

The timing of myometrial activation is central to both physiologic labor and preterm labor risk. During most of gestation, the uterus remains mechanically adaptable while limiting coordinated excitability; at term, the same tissue acquires the electrical, inflammatory, and contractile readiness required for effective labor (Shynlova et al., 2020; Wray and Arrowsmith, 2021). Mechanical load is therefore not a passive background variable. It changes across pregnancy and can alter how endocrine, inflammatory, and smooth-muscle excitability pathways are interpreted (Wray et al., 2015; Arrowsmith, 2023).

A channel-centered view is useful because mechanical force is ultimately translated into ionic flux, membrane potential changes, Ca²^+^ handling, transcriptional programs, inflammatory signaling, and contractile behavior. This does not mean that every labor pathway is an ion-channel pathway, or that every mechanically responsive conductance is a primary mechanosensor. It means that ion channels occupy a strategic interface between tissue loading, cellular excitability, Ca²^+^ entry, and contraction timing.

Existing reviews have clarified myometrial activation (Shynlova et al., 2020), uterine excitability (Wray and Arrowsmith, 2021), and mechanical factors in labor timing (Arrowsmith, 2023). Hormonal control has also been reviewed in detail (Hamburg-Shields and Mesiano, 2024), but a focused synthesis is still needed for human-centered, model-supported ion-channel mechanotransduction in the pregnant myometrium. The scope here is deliberately narrower than broad pregnancy mechanobiology, placenta-first signaling, fetal-membrane biology, cervical remodeling, immune-cell pregnancy biology, or general preterm-birth etiology. Preterm birth is discussed only where the evidence informs premature myometrial activation, preterm labor relevance, or inflammatory and hypercontractile timing problems (Fan et al., 2025).

For clarity, the shorthand used below is defined here: PIEZO1 and PIEZO2 are mechanically activated Piezo channels; TRPV4 is transient receptor potential vanilloid 4; TRPC denotes transient receptor potential canonical channels, with TRPC3 used where a specific isoform is discussed; TREK-1 is a two-pore-domain K^+^ channel; TMEM16A/ANO1 is a Ca²^+^-activated Cl^-^ channel; TRPM4 is transient receptor potential melastatin 4; L-type voltage-gated Ca²^+^ channels, connexin-based gap junctions, and BK/KCa channels are treated as major executors of membrane excitability and contraction propagation rather than as primary stretch sensors.

This is a focused mechanistic narrative Review. Source selection prioritized studies and reviews that inform pregnant myometrium or uterine smooth muscle, PIEZO/TRPV4/TRPC/TREK/TMEM16A/TRPM4 biology, stretch or inflammatory perturbation, human ex vivo tissue evidence, pregnancy model systems, and engineered platforms that clarify mechanical assay context. Evidence from adjacent reproductive tissues is used as boundary or background support rather than as primary evidence for myometrial labor outcomes.

Throughout this Review, “mechanosensitive” is reserved for evidence that directly links mechanical force, stretch, or force-gated channel activity to the tested myometrial context. “Mechanically responsive” describes pathways altered by stretch, matrix, culture mechanics, or tissue architecture without proving direct force gating. “Adjacent excitability regulation” describes conductance and Ca²^+^-handling pathways that shape membrane potential, Ca²^+^ entry, or contractile readiness downstream of, adjacent to, or in parallel with mechanotransduction.

The Review first defines the mechanical and endocrine context needed to interpret channel evidence, then evaluates PIEZO and TRPV4 as the most visible force- and Ca²^+^-linked candidates, places TRPC, TREK, TMEM16A, TRPM4, and related pathways in their functional excitability roles, and finally separates coordinated term parturition from overdistension-associated and infection/inflammation-associated premature activation.

## 2 Mechanical context and evidence framework in the pregnant myometrium

Pregnant myometrium is interpreted through mechanical stretch, progesterone and estrogen balance, oxytocin tone, extracellular-matrix state, tissue architecture, and the gestational transition from quiescence to activation. Uterine stretch and progesterone action have long been linked to labor timing, and modern accounts of myometrial activation emphasize that endocrine and mechanical contexts interact rather than operating as isolated pathways (Lei et al., 2011; Shynlova et al., 2020).

Assay context is therefore part of the evidence, not a technical afterthought. A channel agonist, inhibitor, deletion, or expression change can have different functional meaning in isolated myocytes, engineered tissues, human ex vivo strips, animal pregnancy models, or inflamed tissue. Hydrogel and engineered-tissue systems show that the mechanical microenvironment can alter myometrial morphology, Ca²^+^ responsiveness, gene expression, and agonist response patterns (Maxey et al., 2023; Marr et al., 2024). A scalable hydrogel platform extends this model-system evidence (Claure et al., 2026), but these platforms remain model systems rather than *in vivo* labor-outcome evidence.

Mechanical signals are also distributed through cytoskeletal, adhesion, and phospho-signaling networks. Studies of mechanoadaptation and mechanical strain support a tissue-scale view in which force is interpreted through architecture and intracellular signaling rather than through a single membrane channel (Wu et al., 2008; Copley Salem et al., 2018). Integrin organization adds adhesion-complex context (McLendon et al., 2021). Human labor mechanotransduction models and uterine bioelectrical behavior add complementary tissue-level context (Young, 2016; Garfield et al., 2021).

This Review interprets evidence on three levels: direct force-gated or force-linked channel activation in myometrial systems; mechanically regulated channel expression or signaling without direct proof of force gating; and adjacent excitability pathways that shape the consequences of mechanical, endocrine, or inflammatory inputs. Stretch-induced receptor and adhesion/kinase changes provide important context (Terzidou et al., 2005; Li et al., 2009). Prolonged-stretch contractility evidence adds a separate model boundary (Tattersall et al., 2012). The strength of PIEZO, TRPV4, or TRPC claims therefore depends on channel-specific evidence in the tested system.

## 3 PIEZO1 and PIEZO2: candidate direct mechanosensors with state-dependent directionality

PIEZO channels provide the clearest route for linking mechanical force to myometrial signaling, but their interpretation is best separated by evidence layer. Recent mechanistic work positions PIEZO1 and PIEZO2 as force-sensitive channels involved in parturition-relevant uterine contraction programs in tested models (Zhang et al., 2025). This evidence anchors the stretch-to-channel concept more directly than older general discussions of stretch-activated currents.

Current human myometrial evidence is stronger for PIEZO1 than for PIEZO2. PIEZO1 presence and pharmacologic modulation have been reported in relevant human tissue contexts, but one important ex vivo line points toward Yoda1-associated relaxation and endothelial-myometrial crosstalk rather than a uniform pro-contractile effect (Barnett et al., 2023). In contrast, inflammation-associated preterm-birth models link uterine PIEZO1 overexpression to contraction and inflammatory activation (Bi et al., 2024). These findings indicate compartment- and state-specific effects rather than a single uniform direction of action.

Three interpretive distinctions are central. Genetic or preclinical PIEZO1/PIEZO2 findings, human tissue pharmacology, and inflammatory perturbation studies represent different evidence layers. Endothelial, vascular, and myocyte localization may imply different cellular effects. Agonist-driven, deletion, overexpression, and inflammatory systems may capture different points in a pathway. Human PIEZO1 pharmacology and inflammatory-model findings are therefore interpreted as complementary but non-interchangeable contexts (Barnett et al., 2023; Bi et al., 2024). Parturition-model PIEZO evidence adds model support (Zhang et al., 2025). PIEZO2 is treated more cautiously than PIEZO1 in the human myometrial setting because direct human evidence remains limited.

PIEZO2 also requires a neural boundary. In somatosensory biology, PIEZO2 is a key mechanotransducer for human touch and proprioception (Chesler et al., 2016). The recent parturition study supports a role for PIEZO channels in uterine contraction programs, but direct pregnant-human-myometrium evidence remains much stronger for PIEZO1 than for PIEZO2 (Zhang et al., 2025). A plausible extension is that uterine or cervical stretch could be detected by sensory afferents and coupled to autonomic, neuroendocrine, or local neuromodulatory pathways; catecholaminergic neurofibers in the pregnant cervix provide adjacent reproductive-tract context for neural participation (Malvasi et al., 2024). This neural route is best framed as a mechanistic hypothesis, not as established evidence that PIEZO2 directly gates human myometrial contraction.

Evidence from uterine hypercontractility outside normal pregnancy can support plausibility, but it is treated here as uterine smooth-muscle context rather than as pregnancy-specific evidence. For example, PIEZO1-related hypercontractility in adenomyosis-associated dysmenorrhea supports PIEZO-linked uterine smooth-muscle function, but it does not establish a pregnancy-specific preterm-birth mechanism (Yan et al., 2026).

**Figure 1:**
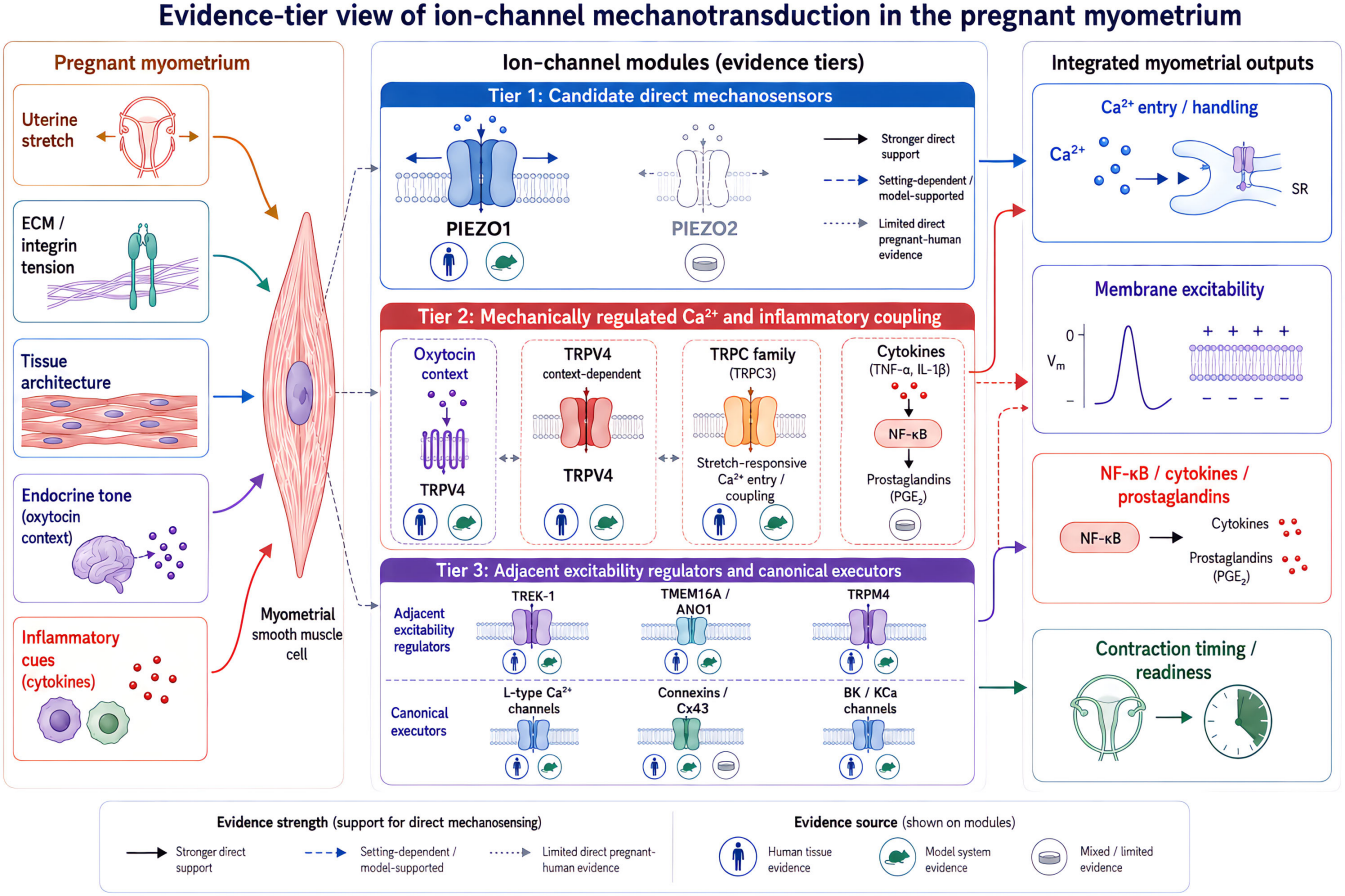
Evidence-tier view of ion-channel mechanotransduction in the pregnant myometrium. The schematic integrates mechanical, endocrine, and inflammatory inputs that can influence ion-channel signaling in the pregnant myometrium. Candidate direct mechanosensor pathways are distinguished from mechanically regulated Ca²^+^/inflammatory coupling pathways and from adjacent excitability regulators. Solid arrows indicate links directly supported in the cited experimental context; dashed arrows indicate setting-dependent or model-supported links; and muted or dotted visual emphasis indicates more limited direct evidence in pregnant human myometrium. PIEZO1 evidence is anchored by human tissue and inflammatory-model studies (Barnett et al., 2023; Bi et al., 2024), while parturition-model evidence extends the PIEZO concept (Zhang et al., 2025). TRPC/TRPV4 evidence supports Ca²^+^/inflammatory coupling in specific contexts (Dalrymple et al., 2007; Ingles et al., 2025), with oxytocin-linked TRPV4 evidence shown separately (Fornes et al., 2026). Adjacent excitability examples include TRPM4 and TMEM16A-related pathways (Fouchet et al., 2023; Parkington et al., 2026). The figure summarizes a bounded synthesis rather than a single validated causal pathway.

The synthesis is therefore not a single arrow from stretch to PIEZO1 to labor. PIEZO channels provide a candidate mechanotransduction route, but PIEZO1 directionality depends on compartment, pregnancy context, perturbation, and inflammatory state; PIEZO2 remains a more limited human-myometrium claim. **Figure 1**, **Table 1** summarize this distinction across evidence layers.

**Table 1:** Channel-specific evidence boundaries in myometrial mechanotransduction.

Channel/module	Human evidence	Functional readout	Interpretation boundary	Representative studies
PIEZO2	Limited direct pregnant-human myometrium evidence; mainly model-supported	Force-linked parturition signaling tested mainly in models	Candidate mechanosensor; human relevance remains tentative	(Zhang et al., 2025)
PIEZO1	Human tissue presence/pharmacology; inflammatory-model support	Ex vivo and inflammatory models capture different states	Compartment- and state-specific directionality	(Barnett et al., 2023; Bi et al., 2024)
TRPV4	Ca²^+^/contractility/inflammation coupling	Oxytocin, NF-κB, tone, and tocolytic contexts	Not a universal stretch sensor or contraction switch	Inflammation/oxytocin: (Ingles et al., 2025; Fornes et al., 2026). Uterine tone: (Ying et al., 2015). Directional boundary: (Villegas et al., 2021)
TRPC family	Stretch-responsive or labor-associated Ca²^+^ entry	Ca²^+^ entry and mechanically responsive signaling where directly tested	TRPC3 is not a primary mechanosensor by default	TRPC stretch/expression: (Dalrymple et al., 2004, 2007). Contractility model: (Chung et al., 2014). TRPC3 axis: (Chen et al., 2018, 2024)
TREK/TMEM16A/TRPM4	Adjacent excitability regulation	K^+^, Cl^-^, and cation conductances shape membrane readiness	Uterine channel function is not equivalent to mechanotransduction-driver status	TREK: (Buxton et al., 2010, 2011). Variant support: (Wu et al., 2012). Adjacent channels: (Heyman et al., 2013; Fouchet et al., 2023). TMEM16A: (Parkington et al., 2026)
Ca²^+^-handling modules	Context-setting regulation	Ca²^+^ homeostasis frames activation state	Indirect Ca²^+^ changes are insufficient to prove direct stretch sensing	SOCE/CD38: (Tribe et al., 2000; Dogan et al., 2024). STIM/Orai: (Chin-Smith et al., 2014). SLO2.1/NALCN: (Ferreira et al., 2025)

## 4 TRPV4: setting-sensitive calcium and inflammatory coupling

TRPV4 is a strong Ca²^+^-linked myometrial signaling candidate, but it is not best interpreted as a universal pro-contractile stretch sensor. In gravid human uterine contexts, TRPV4 has been linked to oxytocin-associated Ca²^+^ entry and smooth-muscle contraction (Fornes et al., 2026). In inflammatory preterm-labor models, TRPV4 loss or inhibition can reduce NF-κB-associated myometrial inflammation and preterm-labor susceptibility (Ingles et al., 2025).

Effect direction is assay-, agonist-, and state-dependent. Human myometrial tissue studies of TRPV4 activation have also reported reduced contractility under tested conditions, which blocks a selective pro-contractile narrative (Villegas et al., 2021). Additional uterine-tone and pregnancy-context evidence supports TRPV4 involvement in contractility, but this remains model- and pharmacology-dependent (Singh et al., 2015; Ying et al., 2015).

Not all TRPV4 evidence is stretch evidence. Oxytocin-associated TRPV4 activation, direct TRPV4 agonism, inflammatory NF-κB signaling, and uterine-tone experiments are distinct contexts. The available evidence does not show that uterine stretch directly activates TRPV4 *in vivo* during labor. Direct stretch/Ca²^+^ evidence is better supported through TRPC pathways (Dalrymple et al., 2007). TRPV4 is used here for inflammatory amplification and oxytocin-associated coupling (Ingles et al., 2025; Fornes et al., 2026).

Regulatory layers further shape this interpretation. miR-203 modulation of the TRPV4-contractility axis and pharmacologic interactions involving calcium-channel or TRPC-related pathways indicate that channel function is embedded in broader transcriptional and Ca²^+^-handling programs (Yart et al., 2022; Ying et al., 2024). These sources support TRPV4 as a state-specific coupling pathway rather than an isolated determinant of labor onset.

Together, these studies support TRPV4 as a state-specific Ca²^+^/inflammatory coupling pathway rather than a universal direct stretch sensor or uniformly pro-contractile pathway in the pregnant human myometrium.

Cross-system PIEZO1-TRPV4 studies help interpret this uncertainty without being directly transferable to the uterus. In pancreatic acinar cells, PIEZO1 activation can open TRPV4 and generate sustained Ca²^+^ signaling during pressure-induced pancreatitis (Swain et al., 2020). In endothelial cells, high shear stress has been reported to place PIEZO1 upstream of TRPV4, with the TRPV4-dependent sustained Ca²^+^ phase contributing to junctional and cytoskeletal changes (Swain and Liddle, 2021). These studies support three uterine hypotheses: PIEZO1 and TRPV4 may act independently in different cell compartments; they may converge on shared Ca²^+^, inflammatory, or contractility outputs; or, in some states, PIEZO1 may lie upstream of TRPV4-like sustained Ca²^+^ signaling. At present, only the first two interpretations are directly defensible for pregnant myometrium; a sequential PIEZO1-to-TRPV4 pathway remains a testable extrapolation rather than a demonstrated labor mechanism.

## 5 Adjacent excitability pathways: TRPC/TREK modules and canonical executors of membrane coupling

The remaining channel pathways are included for their physiologic function, not to create a catalogue of uterine ion channels. Their role is to explain how Ca²^+^, K^+^, Cl^-^, and membrane-excitability pathways, together with canonical voltage-gated and cell-coupling executors, shape the conversion of mechanical, oxytocin-associated, or inflammatory inputs into contractile readiness. General reviews of uterine excitability and electromechanical signaling provide the background for this functional grouping (Wray et al., 2015; Wray and Arrowsmith, 2021).

TRPC channels are most relevant as supporting Ca²^+^-entry pathways. Mechanical stretch can regulate TRPC expression and Ca²^+^ entry in human myometrial smooth-muscle cells, and labor or inflammatory contexts can influence TRPC-related Ca²^+^ pathways (Dalrymple et al., 2004, 2007). Rat and human uterine studies support TRPC or TRP-homologue expression in pregnant myometrium (Dalrymple et al., 2002; Ku et al., 2006). Rat capacitative calcium TrpC protein evidence adds pregnancy-model support (Babich et al., 2004). Stretch-induced uterine contraction models then provide source-specific support for TRPC4/5-like channels in pregnancy-associated contractile responses (Chung et al., 2014).

TRPC3 requires a narrower interpretation. The miR-26a-5p/TRPC3 preterm-labor axis and the PLC-gamma/PKC/CPI-17 pathway support TRPC3 as a preterm-labor-related Ca²^+^ or excitability regulator, but by themselves they provide limited support for TRPC3 as a primary mechanosensor (Chen et al., 2018, 2024). In the current evidence base, TRPC3 is therefore best placed in downstream Ca²^+^-dependent regulation rather than direct stretch sensing.

K^+^ and Cl^-^ pathways provide hyperpolarizing brakes and depolarizing or excitability support. TREK-1 and related stretch-activated potassium currents can shape uterine smooth-muscle contraction and pregnancy-associated excitability (Buxton et al., 2010, 2011). Human variant evidence adds preterm-labor context (Wu et al., 2012). Human myometrial TREK-1 currents and TRPM4 contribute to reproductive-tract and uterine contraction physiology (Heyman et al., 2013; Fouchet et al., 2023). TMEM16A chloride-channel evidence adds a female-reproductive-tract and labor-related excitability boundary (Parkington et al., 2026).

L-type voltage-gated Ca²^+^ channels, connexin/gap-junction coupling, and BK/KCa channels therefore remain explicit in this section. L-type channels convert depolarization into phasic Ca²^+^ entry and contraction, connexin-based coupling permits electrical and contractile propagation across the myometrium, and BK/KCa currents buffer excitability and contribute to quiescence or repolarization. They are central to contraction propagation and tocolytic pharmacology, including nifedipine-linked L-type Ca²^+^-channel blockade, but they function here as downstream or parallel executors of membrane excitability rather than as proof of direct stretch sensing. Broad electromechanical and excitability reviews provide the main executor context (Wray et al., 2015; Wray and Arrowsmith, 2021). Nifedipine/TRPC1 evidence adds a pharmacologic boundary (Yart et al., 2022).

Calcium-handling and homeostatic pathways complete the physiologic context. Store-operated Ca²^+^ entry supports a myometrium in which contraction timing depends on multiple interacting electrical and Ca²^+^ programs (Tribe et al., 2000; Chin-Smith et al., 2014). CD38/cADPR signaling adds another Ca²^+^-handling layer (Dogan et al., 2024). SLO2.1/NALCN activity and oxytocin-related K^+^ conductance regulation add an additional excitability layer (Ferreira et al., 2019, 2025). These pathways support the central argument without replacing channel-specific mechanotransduction evidence.

## 6 Inflammation and distinct routes to term and preterm myometrial activation

**Figure 2:**
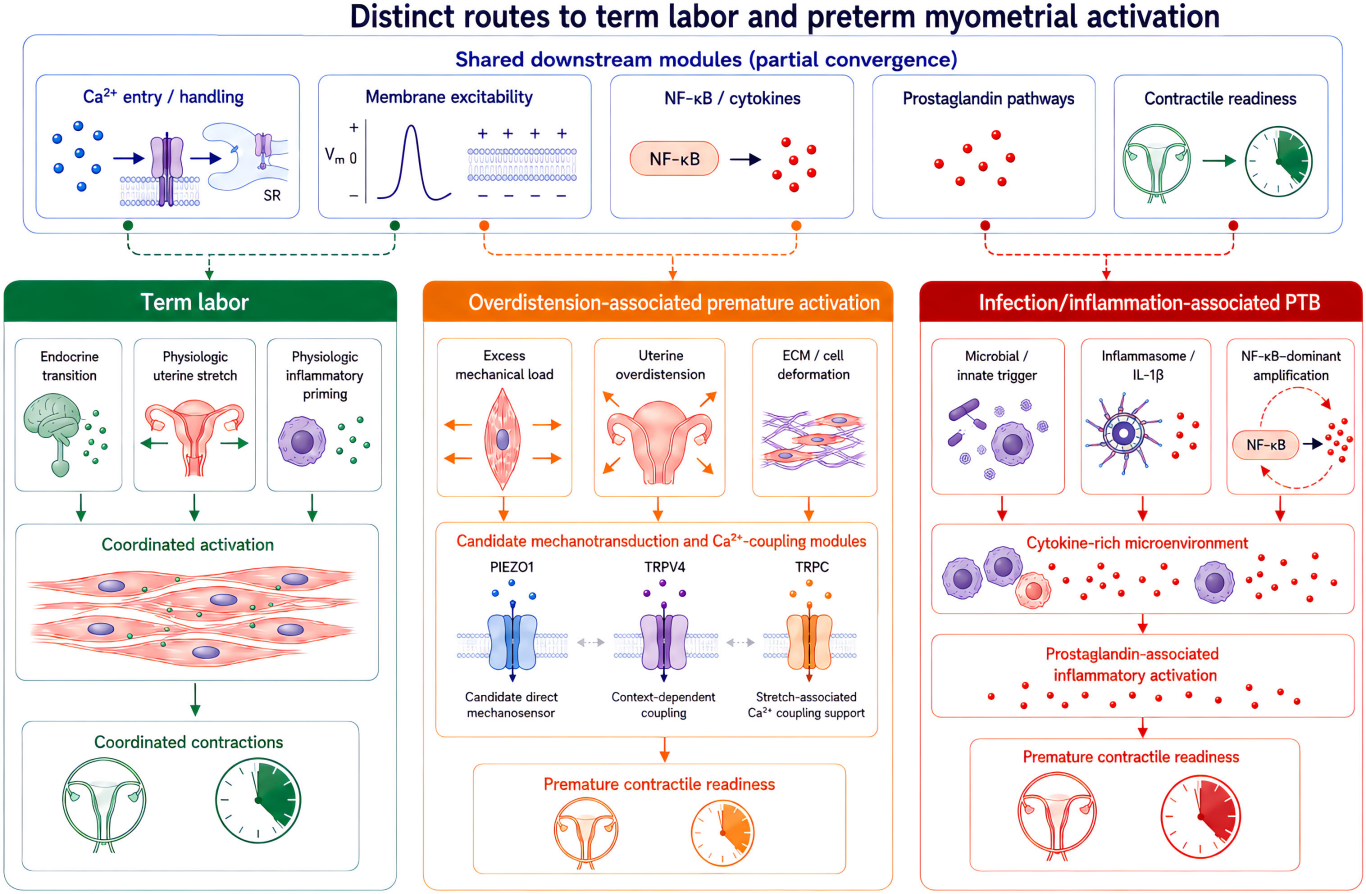
Distinct routes to term labor and preterm myometrial activation. Physiologic term parturition, overdistension-associated premature activation, and infection/inflammation-associated PTB are shown as partially overlapping but mechanistically non-equivalent routes to altered myometrial readiness. Shared downstream modules, including Ca²^+^ signaling, membrane excitability, cytokine/NF-κB activity, prostaglandin pathways, and contractile readiness, still differ in triggers, timing, and tissue-state biology. Term inflammatory priming is supported by NF-κB and cytokine literature (Cookson and Chapman, 2010; Khanjani et al., 2011) and by labor-onset synthesis (Sivarajasingam et al., 2016). Overdistension and stretch-related premature activation are supported by mechanical/endocrine evidence (Shynlova et al., 2008; Lee et al., 2015) and by human/nonhuman-primate overdistension evidence (Adams Waldorf et al., 2015). Infection/inflammation-associated PTB is represented by immune and inflammasome evidence (Lei et al., 2024; Lopez et al., 2024), with infection-related clinical context shown separately (Fan et al., 2025). The figure is limited to evidence synthesis, without treatment, prediction, or one-step causal claims beyond the cited literature.

Inflammation is part of normal labor biology, but it is also a route through which premature activation can be amplified. Myometrial cytokines, NF-κB activity, prostaglandin-related signaling, and endocrine-mechanical integration support inflammatory participation in labor onset and myometrial activation (Khanjani et al., 2011; Sivarajasingam et al., 2016). Myometrial leukocyte interactions add immune-cell context (Siewiera and Erlebacher, 2020). A channel-centered synthesis connects this biology to Ca²^+^ and excitability while preserving differences among triggers. **Figure 2** and **Tables 2**, **3** summarize these distinct routes, their shared downstream modules, and the evidentiary limits of the contributing model systems.

**Table 2:** Distinct trigger contexts that converge on myometrial activation.

Activation context	Principal triggers	Strongest evidence layer	Shared downstream modules	Distinguish from	Representative evidence
Physiologic term activation	Endocrine transition; physiologic inflammatory priming	Predominantly human physiology	Ca²^+^, excitability, cytokine/prostaglandin, readiness	Infection-driven PTB by default	Human inflammatory genes: (Khanjani et al., 2011). Term physiology: (Shynlova et al., 2013; Sivarajasingam et al., 2016)
Stretch/endocrine convergence	Uterine stretch; oxytocin readiness; chemokines	Human tissue + mechanobiology	Mechanical and endocrine convergence	Single linear stretch-to-labor pathway	Stretch/prostaglandin: (Korita et al., 2004; Sooranna et al., 2005). CCL2/MCP-1: (Shynlova et al., 2008). Chemokine support: (Zhao et al., 2013)
Overdistension-associated PTL	Mechanical overload; stretch-cytokine coupling	Human/nonhuman-primate + mechanobiology	Premature activation under load	Infection/inflammasome PTB	(Adams Waldorf et al., 2015; Lee et al., 2015)
Infection/inflammation-associated PTB	Microbial and innate immune activation	Model-dominant + human inflammatory support	Pathological activation before term	Physiologic term priming	Model/review evidence: (Lei et al., 2024; Fan et al., 2025). Inflammasome: (Lopez et al., 2024). Human NF-κB/AP-1 support: (Khanjani et al., 2012)
Adjacent fetal membrane/cervix signals	Boundary tissue cues	Adjacent-tissue context	Background tissue-state signals	Primary myometrial mechanism	(Carvajal et al., 2013; Malvasi et al., 2024)

**Table 3:** What each evidence system can and cannot establish in this field.

Evidence layer	Best use	Cannot establish alone	Representative evidence
Human ex vivo myometrium	Tissue-level contraction and pharmacology	*In vivo* labor timing, safety, or patient selection	Assay platform: (Arrowsmith et al., 2018). Pharmacology examples: (Balki et al., 2025; Aye et al., 2018). Directional pharmacology: (Villegas et al., 2021)
Hydrogel/engineered tissues	Matrix, stiffness, architecture, mechanical-context testing	Direct *in vivo* labor-outcome evidence	Matrix/strain: (Maxey et al., 2023; Marr et al., 2024). Hydrogel: (Claure et al., 2026). Inflammatory/bioprinted systems: (Maxey et al., 2024; Ulrich et al., 2025b)
High-throughput Ca²^+^ and contractility assays	Hypothesis generation and modulator screening	Tissue-level validity, *in vivo* labor timing, safety, or patient selection	(Herington et al., 2015; Ulrich et al., 2025a)
Omics and cellular state maps	State-dependent regulatory modules	Direct channel causality without experimental testing	(Ackerman et al., 2021; Pique-Regi et al., 2022). Inflammatory/prostaglandin states: (Diao et al., 2022; Riaposova et al., 2023)

Mechanical and endocrine signals can converge with inflammatory mediators. Stretch-induced cytokine or prostaglandin responses support mechanical-load/inflammatory convergence in myometrial cells and related tissue signaling (Korita et al., 2004; Sooranna et al., 2005). CCL2/MCP-1 adds endocrine-mechanical integration context (Shynlova et al., 2008). NF-κB and AP-1/IL-8 regulation add inflammatory transcriptional context (Cookson and Chapman, 2010; Khanjani et al., 2012), and stretch-induced cytokine effects on endothelial recruitment show tissue-level immune coupling (Lee et al., 2015).

Three contexts are best kept distinct. Physiologic term inflammatory priming contributes to coordinated labor rather than automatically representing disease. Overdistension-associated premature activation is supported by pregnant-woman and nonhuman-primate evidence for mechanical-overload inflammatory signaling (Adams Waldorf et al., 2015). Infection/inflammation-associated PTB is better supported by microbial/innate immune evidence (Lappas, 2017; Lei et al., 2024) and by inflammasome evidence (Lopez et al., 2024). A broader infection-related PTB synthesis frames the clinical context (Fan et al., 2025). These contexts can share mediators, but they are not interchangeable explanations.

TRPV4 and PIEZO evidence is most informative in this inflammatory discussion when it is linked to explicit context labels. TRPV4 can potentiate NF-κB-mediated inflammatory activation in preterm-labor models, and inflammatory PIEZO1 overexpression can be linked to contraction and preterm-birth contexts (Bi et al., 2024; Ingles et al., 2025). These claims remain mechanistic and model-dependent, with insufficient support for a universal inflammatory PTB pathway or a ready drug target.

This synthesis therefore treats coordinated term parturition, overdistension-associated premature activation, and infection/LPS/inflammasome-associated activation as partially overlapping but non-equivalent routes. Shared downstream modules such as Ca²^+^ entry, NF-κB, cytokine signaling, prostaglandin pathways, and contraction readiness can appear in more than one route, but the triggers, timing, tissue states, and evidence layers remain distinct.

## 7 Physiologic parturition versus premature activation: synthesis across timing contexts

The central synthesis is that the same broad physiologic programs can participate in different timing outcomes. Physiologic term parturition and premature activation both involve excitability, Ca²^+^ signaling, endocrine tone, inflammatory mediators, and contraction programs. Their interpretation depends on timing, trigger, tissue state, and regulatory context. Progesterone and parturition-hormone physiology provide endocrine boundary conditions (Shynlova et al., 2023; Hamburg-Shields and Mesiano, 2024), while oxytocin physiology adds a peripartum signaling context (Uvnäs-Moberg, 2024). Progesterone-receptor and broader electromechanical accounts add developmental and timing context (Wu and DeMayo, 2017; Zangeneh and Hantoushzadeh, 2023).

This timing distinction helps resolve apparent channel conflicts. PIEZO1 need not be uniformly pro-contractile or anti-contractile if human ex vivo relaxation/quiescence signals and inflammation-associated contractile/PTB signals arise from different compartments, agonist contexts, and inflammatory states (Barnett et al., 2023; Bi et al., 2024). Similarly, TRPV4 can be linked to oxytocin-associated contraction and inflammatory activation (Ingles et al., 2025; Fornes et al., 2026), while tocolytic or reduced-contractility evidence blocks a simple pro-contractile story (Villegas et al., 2021). Uterine-tone data add another state-specific layer (Ying et al., 2015).

Adjacent excitability evidence requires the same context separation. TRPC and TRPC3 evidence is separated into stretch-responsive Ca²^+^ entry and preterm-labor regulatory support (Dalrymple et al., 2007; Chen et al., 2024). TRPM4 and CD38/cADPR evidence define contractile and Ca²^+^-handling context (Fouchet et al., 2023; Dogan et al., 2024). TMEM16A evidence adds a chloride-channel and reproductive-tract boundary (Parkington et al., 2026). These pathways are not elevated to master mechanotransducers without direct force-sensing evidence.

The physiologic-versus-pathologic distinction also clarifies the clinical framing. Mechanosensitive and mechanically responsive ion channels may identify vulnerabilities in myometrial activation, but current evidence does not support treating them as ready prevention targets for preterm birth. The evidence is strongest for mechanistic plausibility, tissue and model-system biology, and future assay design; it is weaker for direct clinical intervention, timing, safety, and patient selection.

## 8 Discussion and translational outlook

Several conclusions can be drawn with reasonable confidence from the current evidence. Mechanical context changes how myometrial ion-channel evidence is interpreted, PIEZO1 has stronger human myometrial support than PIEZO2, TRPV4 links Ca²^+^ and inflammatory signaling in a setting-limited way rather than acting as a universal stretch sensor, and TRPC/TREK/TMEM16A/TRPM4-related conductances help set excitability and Ca²^+^ handling. Human ex vivo assays can separate tissue-level pharmacology from direct *in vivo* labor timing (Arrowsmith et al., 2018). Engineered tissues clarify mechanical context and assay readout (Maxey et al., 2023; Claure et al., 2026), while bioprinted models extend this assay logic (Ulrich et al., 2025b). High-throughput Ca²^+^ systems add screening readouts rather than clinical validation (Herington et al., 2015).

Major uncertainties remain unresolved. Human tissue pharmacology can reveal contractility effects that isolated cells may miss, but receptor context, donor state, agonist conditions, and inflammatory state can alter interpretation. Retosiban and oxytocin-linked ex vivo studies illustrate this tissue-pharmacology boundary (Aye et al., 2018; Balki et al., 2025). Oxytocin physiology provides broader peripartum context (Uvnäs-Moberg, 2024). Nifedipine/TRPC1 interactions and TRPV4 reduced-contractility evidence further show that channel-centered mechanisms cannot be translated without dose, tissue-state, and safety validation (Villegas et al., 2021; Yart et al., 2022).

Translation requires a higher evidentiary bar than mechanistic plausibility. Single-cell atlases and calcium-transporter signatures can identify tissue-state axes (Ackerman et al., 2021; Pique-Regi et al., 2022). Inflammatory/prostaglandin systems and RAC1 biology help define validation needs under premature-activation conditions (Diao et al., 2022; Riaposova et al., 2023). Engineered inflammatory systems add assay-level context (Maxey et al., 2024). Matrix metallopeptidase-related contraction evidence adds another state-dependent contraction signal (Ulrich et al., 2025a). These sources are insufficient to replace channel-specific evidence or establish a pregnancy intervention.

Future work can focus on three linked questions. First, how do PIEZO1 and PIEZO2 differ by myometrial compartment, inflammatory state, gestational timing, and species? Second, under which agonist, mechanical, and inflammatory conditions does TRPV4 support contraction, relaxation, or inflammatory amplification? Third, when TRPC, TREK, TMEM16A, TRPM4, and related channels are described as mechanically responsive, what evidence separates direct mechanosensing from downstream excitability support? Progress will require state-resolved experimental designs that combine mechanical variables, inflammatory context, human tissue relevance, and carefully chosen readouts.

## 9 Conclusions

The main advance of this Review is not the proposal of a single labor channel pathway, but a bounded synthesis of how force-sensitive, mechanically responsive, and excitability-regulating ion-channel mechanisms may shape the timing of myometrial activation. PIEZO channels provide the strongest candidate direct mechanotransduction route, with PIEZO1 directionality depending on compartment and inflammatory state and PIEZO2 requiring more cautious human-myometrium interpretation. TRPV4, TRPC, TREK, TMEM16A, TRPM4, and related pathways are best understood as state-limited Ca²^+^, inflammatory, and excitability regulators rather than isolated therapeutic switches. Physiologic term parturition, overdistension-associated premature activation, and infection/inflammation-associated preterm birth can share downstream modules, but they differ in trigger, timing, tissue state, and translational readiness.

The strongest future need is not simply more channel association studies, but state-resolved validation in human myometrium and carefully bounded model systems. Such work will determine which channel-linked mechanisms are direct force sensors, which are mechanically regulated coupling pathways, and which are adjacent excitability regulators that shape the consequences of uterine load and inflammation.

## References

[B1] Ackerman W. E. Buhimschi C. S. Snedden A. Summerfield T. L. Zhao G. Buhimschi I. A. (2021). Molecular signatures of labor and nonlabor myometrium with parsimonious classification from 2 calcium transporter genes. JCI Insight 6 (11), e148425. doi: 10.1172/jci.insight.148425 33945511 PMC8262336

[B2] Adams Waldorf K. M. Singh N. Mohan A. R. Young R. C. Ngo L. Das A. . (2015). Uterine overdistention induces preterm labor mediated by inflammation: observations in pregnant women and nonhuman primates. Am. J. Obstet. Gynecol 213, 830.e1–830.e19. doi: 10.1016/j.ajog.2015.08.028 26284599 PMC4679421

[B3] Arrowsmith S. (2023). Multiple pregnancies, the myometrium and the role of mechanical factors in the timing of labour. Curr. Res. Physiol. 6, 100105. doi: 10.1016/j.crphys.2023.100105 38107788 PMC10724211

[B4] Arrowsmith S. Keov P. Muttenthaler M. Gruber C. W. (2018). Contractility measurements of human uterine smooth muscle to aid drug development. J. Vis. Exp. 56639. doi: 10.3791/56639 29443077 PMC5841565

[B5] Aye I. L. M. H. Moraitis A. A. Stanislaus D. Charnock-Jones D. S. Smith G. C. S. (2018). Retosiban prevents stretch-induced human myometrial contractility and delays labor in cynomolgus monkeys. J. Clin. Endocrinol. Metab. 103, 1056–1067. doi: 10.1210/jc.2017-02195 29293998 PMC5868409

[B6] Babich L. G. Ku C.-Y. Young H. W. J. Huang H. Blackburn M. R. Sanborn B. M. (2004). Expression of capacitative calcium TrpC proteins in rat myometrium during pregnancy. Biol. Reprod. 70, 919–924. doi: 10.1095/biolreprod.103.023325 14627551

[B7] Balki M. Miller L. M. Caliaperumal J. Wang S. Huszti E. Kingdom J. C. (2025). Propranolol and oxytocin-induced contractility in gravid human myometrium: an ex vivo laboratory study. BJOG 132, 1228–1237. doi: 10.1111/1471-0528.18146 40129234 PMC12232598

[B8] Barnett S. D. Asif H. Buxton I. L. O. (2023). Novel identification and modulation of the mechanosensitive Piezo1 channel in human myometrium. J. Physiol. 601, 1675–1690. doi: 10.1113/JP283299 35941750 PMC9905381

[B9] Bi Y. Li H. Diao M. Liu Q. Huang L. Tao Y. . (2024). PIEZO1 overexpression in the uterus contributes to myometrium contraction and inflammation-associated preterm birth. J. Transl. Med. 22, 1140. doi: 10.1186/s12967-024-05978-y 39716206 PMC11667994

[B10] Buxton I. L. O. Heyman N. Wu Y. Barnett S. Ulrich C. (2011). A role of stretch-activated potassium currents in the regulation of uterine smooth muscle contraction. Acta Pharmacol. Sin. 32, 758–764. doi: 10.1038/aps.2011.62 21642947 PMC4009969

[B11] Buxton I. L. O. Singer C. A. Tichenor J. N. (2010). Expression of stretch-activated two-pore potassium channels in human myometrium in pregnancy and labor. PloS One 5, e12372. doi: 10.1371/journal.pone.0012372 20811500 PMC2928262

[B12] Carvajal J. A. Delpiano A. M. Cuello M. A. Poblete J. A. (2013). Mechanical stretch increases brain natriuretic peptide production and secretion in the human fetal membranes. Reprod. Sci. 20, 597–604. doi: 10.1177/1933719112459219 23012317 PMC3713537

[B13] Chen J. Liu T. Cui H. Na Q. Liu S. (2024). MiRNA-26a-5p inhibits preterm labor initiation by targeting and regulating TRPC3 ion channel protein expression. Environ. Toxicol. 39, 357–366. doi: 10.1002/tox.23972 37755144

[B14] Chen J. Zheng D. Cui H. Liu S. Zhang L. Liu C. (2018). Roles and mechanisms of TRPC3 and the PLCγ/PKC/CPI-17 signaling pathway in regulating parturition. Mol. Med. Rep. 17, 898–910. doi: 10.3892/mmr.2017.7998 29115500 PMC5780171

[B15] Chesler A. T. Szczot M. Bharucha-Goebel D. Čeko M. Donkervoort S. Laubacher C. . (2016). The role of PIEZO2 in human mechanosensation. N. Engl. J. Med. 375, 1355–1364. doi: 10.1056/NEJMoa1602812 27653382 PMC5911918

[B16] Chin-Smith E. C. Slater D. M. Johnson M. R. Tribe R. M. (2014). STIM and Orai isoform expression in pregnant human myometrium: a potential role in calcium signaling during pregnancy. Front. Physiol. 5, 169. doi: 10.3389/fphys.2014.00169 24834055 PMC4018559

[B17] Chung S. Kim Y.-H. Joeng J.-H. Ahn D.-S. (2014). Transient receptor potential c4/5 like channel is involved in stretch-induced spontaneous uterine contraction of pregnant rat. Korean J. Physiol. Pharmacol. 18, 503–508. doi: 10.4196/kjpp.2014.18.6.503 25598665 PMC4296040

[B18] Claure I. C. Klapperich C. M. Wong J. Y. (2026). Identifying mechano-modulators of myometrial contractility in a scalable hydrogel culture platform. Tissue Eng. Part A, 19373341261449868. 42175906 10.1177/19373341261449868

[B19] Cookson V. J. Chapman N. R. (2010). NF-kappaB function in the human myometrium during pregnancy and parturition. Histol. Histopathol. 25, 945–956. doi: 10.14670/HH-25.945 20503182

[B20] Copley Salem C. Ulrich C. Quilici D. Schlauch K. Buxton I. L. O. Burkin H. (2018). Mechanical strain induced phospho-proteomic signaling in uterine smooth muscle cells. J. Biomech. 73, 99–107. doi: 10.1016/j.jbiomech.2018.03.040 29661501 PMC5932261

[B21] Dalrymple A. Mahn K. Poston L. Songu-Mize E. Tribe R. M. (2007). Mechanical stretch regulates TRPC expression and calcium entry in human myometrial smooth muscle cells. Mol. Hum. Reprod. 13, 171–179. doi: 10.1093/molehr/gal110 17208928

[B22] Dalrymple A. Slater D. M. Beech D. Poston L. Tribe R. M. (2002). Molecular identification and localization of Trp homologues, putative calcium channels, in pregnant human uterus. Mol. Hum. Reprod. 8, 946–951. doi: 10.1093/molehr/8.10.946 12356946

[B23] Dalrymple A. Slater D. M. Poston L. Tribe R. M. (2004). Physiological induction of transient receptor potential canonical proteins, calcium entry channels, in human myometrium: influence of pregnancy, labor, and interleukin-1 beta. J. Clin. Endocrinol. Metab. 89, 1291–1300. doi: 10.1210/jc.2003-031428 15001625

[B24] Diao M. Zhou J. Tao Y. Hu Z. Lin X. (2022). RAC1 is involved in uterine myometrium contraction in the inflammation-associated preterm birth. Reproduction 164, 169–181. doi: 10.1530/REP-21-0186 36018772 PMC9513643

[B25] Dogan S. Walseth T. F. Guvenc Tuna B. Uçar E. Kannan M. S. Deshpande D. A. (2024). CD38/cADPR-mediated calcium signaling in a human myometrial smooth muscle cell line, PHM1. IUBMB Life 76, 1223–1233. doi: 10.1002/iub.2904 39135342 PMC11580371

[B26] Fan S. Li Q. Feng Q. Zhao P. Zhang X. (2025). Infection-related preterm birth. Matern Fetal Med. 7, 172–180. doi: 10.1097/FM9.0000000000000288 41608213 PMC12846865

[B27] Ferreira J. J. Butler A. Stewart R. Gonzalez-Cota A. L. Lybaert P. Amazu C. . (2019). Oxytocin can regulate myometrial smooth muscle excitability by inhibiting the Na+ -activated K+ channel, Slo2.1. J. Physiol. 597, 137–149. doi: 10.1113/JP276806 30334255 PMC6312452

[B28] Ferreira J. J. Kent L. N. McCarthy R. Butler A. Ma X. Peramsetty N. . (2025). SLO2.1/NALCN functional complex activity in mouse myometrial smooth muscle cells during pregnancy. J. Cell. Physiol. 240, e70024. doi: 10.1002/jcp.70024 40170408 PMC11962242

[B29] Fornes D. Ansari J. R. Michel G. Alvira C. M. Cornfield D. N. (2026). In the gravid human uterus, oxytocin induces smooth muscle cell contraction via transient receptor potential vanilloid 4 channel activation. J. Physiol. 604, 3135–3158. doi: 10.1113/JP289262 41762213

[B30] Fouchet A. Bangando H. M. Aize M. Simard C. Guinamard R. (2023). TRPM4 contribution in mouse uterine contractions. Reproduction 166, 77–87. doi: 10.1530/REP-22-0484 37204208

[B31] Garfield R. E. Murphy L. Gray K. Towe B. (2021). Review and study of uterine bioelectrical waveforms and vector analysis to identify electrical and mechanosensitive transduction control mechanisms during labor in pregnant patients. Reprod. Sci. 28, 838–856. doi: 10.1007/s43032-020-00358-5 33090378

[B32] Hamburg-Shields E. Mesiano S. (2024). The hormonal control of parturition. Physiol. Rev. 104, 1121–1145. doi: 10.1152/physrev.00019.2023 38329421 PMC11380996

[B33] Herington J. L. Swale D. R. Brown N. Shelton E. L. Choi H. Williams C. H. . (2015). High-throughput screening of myometrial calcium-mobilization to identify modulators of uterine contractility. PloS One 10, e0143243. doi: 10.1371/journal.pone.0143243 26600013 PMC4658040

[B34] Heyman N. S. Cowles C. L. Barnett S. D. Wu Y.-Y. Cullison C. Singer C. A. . (2013). TREK-1 currents in smooth muscle cells from pregnant human myometrium. Am. J. Physiol. Cell Physiol. 305, C632–C642. doi: 10.1152/ajpcell.00324.2012 23804201 PMC3761174

[B35] Ingles J. A. Rodriguez Z. Fornes D. Ying L. Han X. Cornfield D. N. . (2025). Loss of TRPV4 decreases NFκB-mediated myometrial inflammation and prevents preterm labor. FASEB J. 39, e70418. doi: 10.1096/fj.202402949R 39989412

[B36] Khanjani S. Kandola M. K. Lindstrom T. M. Sooranna S. R. Melchionda M. Lee Y. S. . (2011). NF-κB regulates a cassette of immune/inflammatory genes in human pregnant myometrium at term. J. Cell. Mol. Med. 15, 809–824. doi: 10.1111/j.1582-4934.2010.01069.x 20406326 PMC3922669

[B37] Khanjani S. Terzidou V. Johnson M. R. Bennett P. R. (2012). NFκB and AP-1 drive human myometrial IL8 expression. Mediators Inflamm. 2012, 504952. doi: 10.1155/2012/504952 22685373 PMC3364596

[B38] Korita D. Itoh H. Sagawa N. Yura S. Yoshida M. Kakui K. . (2004). Cyclic mechanical stretching and interleukin-1alpha synergistically up-regulate prostacyclin secretion in cultured human uterine myometrial cells. Gynecol Endocrinol. 18, 130–137. doi: 10.1080/09513590410001667850 15255281

[B39] Ku C.-Y. Babich L. Word R. A. Zhong M. Ulloa A. Monga M. . (2006). Expression of transient receptor channel proteins in human fundal myometrium in pregnancy. J. Soc Gynecol Investig. 13, 217–225. doi: 10.1016/j.jsgi.2005.12.007 16527499

[B40] Lappas M. (2017). The IL-1β signalling pathway and its role in regulating pro-inflammatory and pro-labour mediators in human primary myometrial cells. Reprod. Biol. 17, 333–340. doi: 10.1016/j.repbio.2017.09.006 28988892

[B41] Lee Y.-H. Shynlova O. Lye S. J. (2015). Stretch-induced human myometrial cytokines enhance immune cell recruitment via endothelial activation. Cell. Mol. Immunol. 12, 231–242. doi: 10.1038/cmi.2014.39 24882387 PMC4654292

[B42] Lei K. Chen L. Cryar B. J. Hua R. Sooranna S. R. Brosens J. J. . (2011). Uterine stretch and progesterone action. J. Clin. Endocrinol. Metab. 96, E1013–E1024. doi: 10.1210/jc.2010-2310 21450990

[B43] Lei W.-J. Zhang F. Li M.-D. Pan F. Ling L.-J. Lu J.-W. . (2024). C/EBPδ deficiency delays infection-induced preterm birth. BMC Med. 22, 432. doi: 10.1186/s12916-024-03650-2 39379940 PMC11462803

[B44] Li Y. Reznichenko M. Tribe R. M. Hess P. E. Taggart M. Kim H. . (2009). Stretch activates human myometrium via ERK, caldesmon and focal adhesion signaling. PloS One 4, e7489. doi: 10.1371/journal.pone.0007489 19834610 PMC2759504

[B45] Lopez T. E. Zhang H. Bouysse E. Neiers F. Ye X. Y. Garrido C. . (2024). A pivotal role for the IL-1β and the inflammasome in preterm labor. Sci. Rep. 14, 4234. doi: 10.1038/s41598-024-54507-w 38378749 PMC10879161

[B46] Malvasi A. Baldini G. M. Cicinelli E. Di Naro E. Baldini D. Favilli A. . (2024). Localization of catecholaminergic neurofibers in pregnant cervix as a possible myometrial pacemaker. Int. J. Mol. Sci. 25 (11), 5630. doi: 10.3390/ijms25115630 38891818 PMC11171499

[B47] Marr E. E. Isenberg B. C. Wong J. Y. (2024). Effects of polydimethylsiloxane membrane surface treatments on human uterine smooth muscle cell strain response. Bioact. Mater. 32, 415–426. doi: 10.1016/j.bioactmat.2023.10.006 37954466 PMC10632608

[B48] Maxey A. P. Travis J. M. McCain M. L. (2023). Regulation of oxytocin-induced calcium transients and gene expression in engineered myometrial tissues by tissue architecture and matrix rigidity. Curr. Res. Physiol. 6, 100108. doi: 10.1016/j.crphys.2023.100108 38107790 PMC10724203

[B49] Maxey A. P. Wheeler S. J. Travis J. M. McCain M. L. (2024). Contractile responses of engineered human myometrium to prostaglandins and inflammatory cytokines. APL Bioeng. 8, 046115. doi: 10.1063/5.0233737 39734362 PMC11672207

[B50] McLendon B. A. Kramer A. C. Seo H. Bazer F. W. Burghardt R. C. Johnson G. A. (2021). Integrin adhesion complex organization in sheep myometrium reflects changing mechanical forces during pregnancy and postpartum. Biol. (Basel) 10 (6), 508. doi: 10.3390/biology10060508 34201059 PMC8227588

[B51] Parkington H. C. Coleman H. A. Sheehan P. M. Brennecke S. P. Tare M. (2026). TMEM16A chloride channels in the female reproductive tract and their role in normal and dysfunctional pregnancy and labour. J. Physiol. doi: 10.1113/JP287611 42126110

[B52] Pique-Regi R. Romero R. Garcia-Flores V. Peyvandipour A. Tarca A. L. Pusod E. . (2022). A single-cell atlas of the myometrium in human parturition. JCI Insight 7(5), e153921. doi: 10.1172/jci.insight.153921 35260533 PMC8983148

[B53] Riaposova L. Kim S. H. Hanyaloglu A. C. Sykes L. MacIntyre D. A. Bennett P. R. . (2023). Prostaglandin F2α requires activation of calcium-dependent signalling to trigger inflammation in human myometrium. Front. Endocrinol. (Lausanne) 14, 1150125. doi: 10.3389/fendo.2023.1150125 37547305 PMC10400332

[B54] Shynlova O. Lee Y.-H. Srikhajon K. Lye S. J. (2013). Physiologic uterine inflammation and labor onset: integration of endocrine and mechanical signals. Reprod. Sci. 20, 154–167. doi: 10.1177/1933719112446084 22614625

[B55] Shynlova O. Nadeem L. Lye S. (2023). Progesterone control of myometrial contractility. J. Steroid Biochem. Mol. Biol. 234, 106397. doi: 10.1016/j.jsbmb.2023.106397 37683774

[B56] Shynlova O. Nadeem L. Zhang J. Dunk C. Lye S. (2020). Myometrial activation: Novel concepts underlying labor. Placenta 92, 28–36. doi: 10.1016/j.placenta.2020.02.005 32056784

[B57] Shynlova O. Tsui P. Dorogin A. Lye S. J. (2008). Monocyte chemoattractant protein-1 (CCL-2) integrates mechanical and endocrine signals that mediate term and preterm labor. J. Immunol. 181, 1470–1479. doi: 10.4049/jimmunol.181.2.1470 18606702

[B58] Siewiera J. Erlebacher A. (2020). Myometrial leukocytes. Curr. Opin. Physiol. 13, 6–13. doi: 10.1016/j.cophys.2019.08.004 40809840 PMC12346145

[B59] Singh V. Ram M. Kandasamy K. Thangamalai R. Choudhary S. Dash J. R. . (2015). Molecular and functional characterization of TRPV4 channels in pregnant and nonpregnant mouse uterus. Life Sci. 122, 51–58. doi: 10.1016/j.lfs.2014.12.010 25529150

[B60] Sivarajasingam S. P. Imami N. Johnson M. R. (2016). Myometrial cytokines and their role in the onset of labour. J. Endocrinol. 231, R101–R119. doi: 10.1530/JOE-16-0157 27647860

[B61] Sooranna S. R. Engineer N. Loudon J. A. Z. Terzidou V. Bennett P. R. Johnson M. R. (2005). The mitogen-activated protein kinase dependent expression of prostaglandin H synthase-2 and interleukin-8 messenger ribonucleic acid by myometrial cells: the differential effect of stretch and interleukin-1{beta}. J. Clin. Endocrinol. Metab. 90, 3517–3527. doi: 10.1210/jc.2004-1390 15784717

[B62] Swain S. M. Liddle R. A. (2021). PIEZO1 acts upstream of TRPV4 to induce pathological changes in endothelial cells due to shear stress. J. Biol. Chem. 296, 100171. doi: 10.1074/jbc.RA120.015059 33298523 PMC7948745

[B63] Swain S. M. Romac J. M. Shahid R. A. Pandol S. J. Liedtke W. Vigna S. R. . (2020). TRPV4 channel opening mediates pressure-induced pancreatitis initiated by PIEZO1 activation. J. Clin. Invest. 130, 2527–2541. doi: 10.1172/JCI134111 31999644 PMC7190979

[B64] Tattersall M. Cordeaux Y. Charnock-Jones D. S. Smith G. C. S. (2012). Expression of gastrin-releasing peptide is increased by prolonged stretch of human myometrium, and antagonists of its receptor inhibit contractility. J. Physiol. 590, 2081–2093. doi: 10.1113/jphysiol.2012.228239 22411014 PMC3447152

[B65] Terzidou V. Sooranna S. R. Kim L. U. Thornton S. Bennett P. R. Johnson M. R. (2005). Mechanical stretch up-regulates the human oxytocin receptor in primary human uterine myocytes. J. Clin. Endocrinol. Metab. 90, 237–246. doi: 10.1210/jc.2004-0277 15494465

[B66] Tribe R. M. Moriarty P. Poston L. (2000). Calcium homeostatic pathways change with gestation in human myometrium. Biol. Reprod. 63, 748–755. doi: 10.1095/biolreprod63.3.748 10952916

[B67] Ulrich C. C. Parker L. L. Lambert J. A. Baldwin L. Buxton I. L. O. Etezadi-Amoli N. . (2025a). Matrix metallopeptidase 9 promotes contraction in human uterine myometrium. Reprod. Sci. 32, 444–454. doi: 10.1007/s43032-024-01778-3 39776427 PMC11825266

[B68] Ulrich C. Siddiqui K. Baldwin L. K. Hua W. Kuklok J. K. Okaikoi J. J. . (2025b). A bioprinted model of pregnant human uterine myometrium. Front. Bioeng. Biotechnol. 13, 1632320. doi: 10.3389/fbioe.2025.1632320 41245630 PMC12611874

[B69] Uvnäs-Moberg K. (2024). The physiology and pharmacology of oxytocin in labor and in the peripartum period. Am. J. Obstet. Gynecol 230, S740–S758. doi: 10.1016/j.ajog.2023.04.011 38462255

[B70] Villegas D. Giard O. Brochu-Gaudreau K. Rousseau É. (2021). Activation of TRPV4 channels leads to a consistent tocolytic effect on human myometrial tissues. Eur. J. Obstet. Gynecol Reprod. Biol. X 10, 100124. doi: 10.1016/j.eurox.2021.100124 33733088 PMC7941160

[B71] Wray S. Arrowsmith S. (2021). Uterine excitability and ion channels and their changes with gestation and hormonal environment. Annu. Rev. Physiol. 83, 331–357. doi: 10.1146/annurev-physiol-032420-035509 33158376

[B72] Wray S. Burdyga T. Noble D. Noble K. Borysova L. Arrowsmith S. (2015). Progress in understanding electro-mechanical signalling in the myometrium. Acta Physiol. (Oxf) 213, 417–431. doi: 10.1111/apha.12431 25439280

[B73] Wu S.-P. DeMayo F. J. (2017). Progesterone receptor signaling in uterine myometrial physiology and preterm birth. Curr. Top. Dev. Biol. 125, 171–190. doi: 10.1016/bs.ctdb.2017.03.001 28527571 PMC5542013

[B74] Wu X. Morgan K. G. Jones C. J. Tribe R. M. Taggart M. J. (2008). Myometrial mechanoadaptation during pregnancy: implications for smooth muscle plasticity and remodelling. J. Cell. Mol. Med. 12, 1360–1373. doi: 10.1111/j.1582-4934.2008.00306.x 18363833 PMC2729593

[B75] Wu Y.-Y. Singer C. A. Buxton I. L. O. (2012). Variants of stretch-activated two-pore potassium channel TREK-1 associated with preterm labor in humans. Biol. Reprod. 87, 96. doi: 10.1095/biolreprod.112.099499 22811574 PMC3507547

[B76] Yan D. Wang Y. Liu X. Guo S.-W. (2026). Impaired PIEZO1 function drives uterine hypercontractility in adenomyosis-associated dysmenorrhea. Hum. Reprod. Open 2026, hoag013. doi: 10.1093/hropen/hoag013 41836349 PMC12981915

[B77] Yart L. Frieden M. Konig S. Cohen M. Martinez de Tejada B. (2022). Dual effect of nifedipine on pregnant human myometrium contractility: Implication of TRPC1. J. Cell. Physiol. 237, 1980–1991. doi: 10.1002/jcp.30666 34988986 PMC9306527

[B78] Ying L. Becard M. Lyell D. Han X. Shortliffe L. Husted C. I. . (2015). The transient receptor potential vanilloid 4 channel modulates uterine tone during pregnancy. Sci. Transl. Med. 7, 319ra204. doi: 10.1126/scitranslmed.aad0376 26702092

[B79] Ying L. Fornes D. D. Dobberfuhl A. D. Ansari J. R. Alvira C. M. Cornfield D. N. (2024). miR-203 modulates pregnant myometrium contractility via transient receptor potential vanilloid 4 channel expression. FASEB J. 38, e70173. doi: 10.1096/fj.202401783RR 39545721

[B80] Young R. C. (2016). Mechanotransduction mechanisms for coordinating uterine contractions in human labor. Reproduction 152, R51–R61. doi: 10.1530/REP-16-0156 27165050

[B81] Zangeneh F. Z. Hantoushzadeh S. (2023). The physiological basis with uterine myometrium contractions from electro-mechanical/hormonal myofibril function to the term and preterm labor. Heliyon 9, e22259. doi: 10.1016/j.heliyon.2023.e22259 38034762 PMC10687101

[B82] Zhang Y. Kini S. A. Mishkanian S. A. Yarishkin O. Luo R. Seradj S. H. . (2025). PIEZO channels link mechanical forces to uterine contractions in parturition. Science 390, eady3045. doi: 10.1126/science.ady3045 41231991 PMC12807505

[B83] Zhao Y. Koga K. Osuga Y. Izumi G. Takamura M. Harada M. . (2013). Cyclic stretch augments production of neutrophil chemokines and matrix metalloproteinase-1 in human uterine smooth muscle cells. Am. J. Reprod. Immunol. 69, 240–247. doi: 10.1111/aji.12061 23253141

